# Supplementary material for: HIVprotI: an integrated web based platform for prediction and design of HIV proteins inhibitors
Source: J Cheminform. 2018 Mar 9;10:12. doi: 10.1186/s13321-018-0266-y (PMC5845081; doi:10.1186/s13321-018-0266-y)
Supplement: Supplementary file 2 — Additional file 2. Source code of HIVProtI web server. [file 13321_2018_266_MOESM2_ESM.zip › HIVprotI_Source-code/hivprotI/noslides.html]

vertex - Free CSS Template by ZyPOP


# Website**Name**

## Your website slogan here

- Home
- Examples
  - Static Text Page
  - Static Frontpage
  - Another link
- Products
  - Product One
  - Product Two
  - Product Three
- Solutions
- Contact

## Slide #1

Lorem ipsum dolor sit amet, consectetuer adipiscing elit. Pellentesque venenatis sagittis enim. Maecenas ligula erat, egestas congue, varius nec, sagittis nec, purus. In neque.

Lorem ipsum dolor

## Introduction

Welcome to widget, a free premium valid CSS & XHTML strict web template from spyka Webmaster. This template is completely **free** to use permitting a link remains back to http://www.spyka.net. Should you wish to use this template unbranded you can buy a template license from our website for 8.00 GBP, this will allow you remove all branding related to our site, for more information about this see below.

This template has been tested in:

- Firefox 3.5
- Opera 10.00
- IE 6, 7 and 8
- Chrome

### Buy unbranded

Purchasing a template license for 8.00 GBP (at time of writing around 12 USD) gives you the right to remove any branding including links, logos and source tags relating to spyka webmaster. As well as waiving the attribution requirement, your payment will also help us provide continued support for users as well as creating new web templates. Find out more about how to buy at the licensing page on our website which can be accessed at http://www.spyka.net/licensing

### More free web templates

Looking for more free web templates for other projects? Check out our free web template portfolio. We also offer WordPress themes and phpBB3 styles, all of which are released under Open Source or Creative Commons licenses!

### Webmaster forums

You can get help with editing and using this template, as well as design tips, tricks and advice in our webmaster forums

- #### Our **Pages**

  - Home
  - Examples
  - Products
  - Solutions
  - Contact
- #### About **Us**

  - Aenean nec massa a tortor auctor sodales sed a dolor. Duis vitae lorem sem. Proin at velit vel arcu pretium luctus.
- #### Cool **Sites**

  - **ThemeForest** - premium HTML templates, WordPress themes and PHP scripts
  - **Web hosting** - 50 dollars off when you use promocode **awesome50**
  - **4templates** - brilliant premium templates

#### About our site

Morbi fermentum, nunc id pellentesque blandit, lectus velit pellentesque nisl, a condimentum est velit sed nisi. Sed libero velit, eleifend nec porttitor a, porta quis leo. In hac habitasse platea dictumst.

#### Categories

- Lorem ipsum dolor sit amet.
- Quisque consequat nunc a felis.
- Suspendisse consequat magna at.
- Etiam eget diam id ligula rhoncus.
- Sed in mauris non nibh.

#### Network sites

- ZyPOP web templates
- Free web templates
- SMF themes
- MyBB themes
- phpBB3 styles

#### Search our site

© YourSite 2013. Free CSS Layouts by ZyPOP
